# Electrospun Nanofibers in Wound Healing: Real-World Evaluation of Spincare™ Technology

**DOI:** 10.3390/bioengineering12050500

**Published:** 2025-05-09

**Authors:** Borza Ioan Lucian, Cornel Dragos Cheregi, Horgos Maur Sebastian, Bodog Ruxandra-Florina, Laura Maghiar, Brihan Ilarie, Huniadi Anca, Liliana Sachelarie, Sandor Mircea-Ioan

**Affiliations:** 1 Department of Surgical Disciplines, Faculty of Medicine and Pharmacy, University of Oradea, 1st December Square 10, 410073 Oradea, Romania; borzaioanlucian@yahoo.com (B.I.L.); cornel.cheregi@yahoo.com (C.D.C.); bodogruxandra@gmail.com (B.R.-F.); lauratodan@yahoo.com (L.M.); ancahuniadi@gmail.com (H.A.); drims75@yahoo.com (S.M.-I.); 2 CF Oradea Clinical Hospital, Str. Republic 56, 410159 Oradea, Romania; 3 Avram Iancu Military Hospital, Str. Dunarea nr.3, 410027 Oradea, Romania; 4 Bihor County Emergency Clinical Hospital, Gheorghe Doja Street nr. 57, 410169 Oradea, Romania; brihan_drm@yahoo.com; 5 Psiho-Neuro Sciences Department, Faculty of Medicine and Pharmacy, University of Oradea, 1st December Square 10, 410073 Oradea, Romania; 6 Pelican Clinical Hospital Oradea, Str. Corneliu Coposu nr.14A-14B, 410450 Oradea, Romania; 7 Preclinical Sciences Department, Faculty of Medicine, Apollonia University, 700511 Iasi, Romania

**Keywords:** wound, nanofiber, healing

## Abstract

(1) Background: The increasing prevalence of chronic wounds, along with their significant healthcare burden, underscores the need for innovative and technologically advanced treatment strategies. Electrospun nanofiber-based dressings have emerged as a promising solution, mimicking the skin’s extracellular matrix and promoting efficient tissue regeneration. (2) Methods: This real-world, 10-month observational study conducted at CF Oradea Clinical Hospital enrolled 60 patients with chronic, non-healing wounds. Patients were randomly assigned to two groups: 30 received standard vacuum-assisted wound therapy, serving as the control group. In contrast, 30 received treatment with Spincare™, a novel electrospinning technology that delivers a personalized nanofiber matrix directly onto the wound. Symptom progression, pain levels, and treatment adaptation were assessed using standardized questionnaires. (3) Results: Patients treated with Spincare™ demonstrated faster wound healing, especially in the epithelialization phase, with significantly improved pain scores and quality of life measures. The technology was well-tolerated and reduced the need for repeated hospitalizations. (4) Conclusions: Spincare™ represents an effective and innovative electrospun nanofiber solution for chronic wound management, accelerating healing and enhancing patient outcomes, particularly in individuals with underlying conditions such as peripheral arterial disease. These findings support the integration of electrospinning-based therapies in modern wound care protocols.

## 1. Introduction

Chronic wounds remain a persistent clinical and economic burden, often resisting conventional therapeutic approaches and significantly affecting patients’ quality of life and functional independence [1,2,3]. The associated healthcare costs are substantial, with extended healing times, frequent hospitalizations, and recurring complications representing major cost drivers, far beyond the cost of dressings alone [3,4,5,6,7]. In this context, the need for advanced and efficient wound care strategies is more critical than ever, especially in patients with comorbidities such as diabetes, peripheral arterial disease, or chronic venous insufficiency, where impaired cellular mechanisms hinder the natural healing process [1,2].

Electrospun nanofibers create a highly porous, extracellular matrix-like scaffold that promotes cell adhesion, migration, and proliferation, accelerating wound healing, reducing infection risk, and improving patient outcomes [6]. Spincare™ technology advances this approach by utilizing a portable electrospinning device to apply a personalized nanofiber matrix directly onto the wound surface.

Electrospun nanofibers have gained significant attention in biomedical applications due to their structural similarity to the native extracellular matrix (ECM). These nanofibrous scaffolds provide a biomimetic environment that supports key regenerative processes such as cell adhesion, proliferation, and migration. In wound healing, their high surface area and porosity facilitate moisture retention, oxygen exchange, and exudate management, while also acting as an effective barrier against microbial contamination. Moreover, nanofiber dressings can be functionalized with bioactive molecules, including antimicrobials, growth factors, and anti-inflammatory agents, enhancing their therapeutic potential. Their flexibility and adaptability suit irregular wound surfaces and hard-to-treat chronic wounds. The clinical implementation of this technology, as seen in devices like Spincare™, reflects its successful translation from experimental platforms to real-world patient care, providing tailored scaffolds that actively promote regeneration in complex wound environments [6,7,8,9,10].

Recent advances in tissue engineering and regenerative medicine have opened up promising pathways for managing chronic wounds. Electrospinning technology has gained significant traction for its ability to fabricate electrospun nanofibers that closely replicate the architecture of the skin’s extracellular matrix [4,5,6,7,8]. This structural biomimicry is key in promoting cellular adhesion, proliferation, and migration, all essential processes in wound regeneration [9,10,11].

The electrospinning process is influenced by various interdependent parameters that significantly affect the resulting nanofibers’ morphology, porosity, and mechanical properties. These include solution properties (polymer concentration, molecular weight, viscosity, conductivity, and surface tension), processing conditions (applied voltage, flow rate, distance between the needle and collector), and ambient factors such as temperature and relative humidity. Fine-tuning these variables is essential to ensure uniform fiber formation and optimize their biomedical performance, particularly in wound healing [12,13].

Spincare™ is a novel medical device utilizing portable electrospinning technology, enabling the direct application of a skin-like nanofiber matrix to the wound surface. The process involves transforming a polymeric solution into ultrafine fibers under the influence of a high-voltage electric field. As the polymer jet travels a defined distance, solvent evaporation occurs, and solidified nanofibers are deposited as a uniform layer on the wound bed. Key parameters—such as polymer type, viscosity, molecular weight, voltage, ambient humidity, and collection distance—can be modulated to optimize fiber characteristics and performance [9,10]. Unlike traditional dressings, electrospun nanofiber dressings exhibit high porosity and controlled permeability, enabling the maintenance of moisture balance, regulation of exudate, and gas exchange, all while acting as a temporary skin substitute [6]. The result creates a protective and bioactive environment that accelerates re-epithelialization and tissue regeneration.

The clinical integration of such electrospun nanofiber-based therapies represents a paradigm shift toward personalized, patient-adapted wound care solutions. Our study aimed to evaluate the efficacy of Spincare™ technology in a real-world hospital setting, investigating its performance in terms of healing acceleration, pain control, and patient adaptation compared to conventional vacuum-assisted therapy.

## 2. Materials and Methods

### 2.1. Study Design

This observational study was conducted over 10 months, from 1 October 2022, to 30 June 2023, at the Surgical Clinic of CF Oradea Clinical Hospital. A total of 60 inpatients with non-healing wounds secondary to underlying chronic conditions were enrolled. The study protocol was approved by the Ethics Committee of the Surgical Clinic (approval no. 5518/27.09.2022) and conducted following the ethical standards outlined in the Declaration of Helsinki.

This prospective, real-world observational study included 60 patients with chronic, non-healing wounds due to diabetes, peripheral arterial disease, or chronic venous insufficiency. Patients were randomized into two equal groups: a control group (n = 30) treated with standard negative pressure wound therapy (NPWT) followed by conventional care, and a study group (n = 30) treated with NPWT followed by Spincare™ electrospun nanofiber application. Outcomes were assessed through clinical wound evaluations, patient-reported questionnaires, and histopathological analysis of punch biopsies taken at admission and discharge.

Inclusion criteria comprised adult patients (aged 40–70 years) with chronic wounds related to diabetes mellitus, chronic venous insufficiency, or peripheral arterial disease of the lower limbs who had not previously received advanced wound healing interventions.

Exclusion criteria included patients with superinfected wounds (as confirmed by negative cultures), chronic traumatic wounds, severe immunosuppression (e.g., HIV/AIDS, advanced malignancies, end-stage renal or hepatic disease), cognitive or behavioral limitations impeding treatment compliance, and a lack of informed consent.

### 2.2. Symptom Assessment via Questionnaire

At admission, each patient received a structured questionnaire designed to assess the subjective experience of symptoms and track changes throughout the hospitalization. The questionnaire included numerical rating scales (ranging from 0 to 10) to assess pain intensity, daily discomfort, general condition compared to the admission day, and the patient’s ability to adapt to the applied treatment.

**Figure 1:**
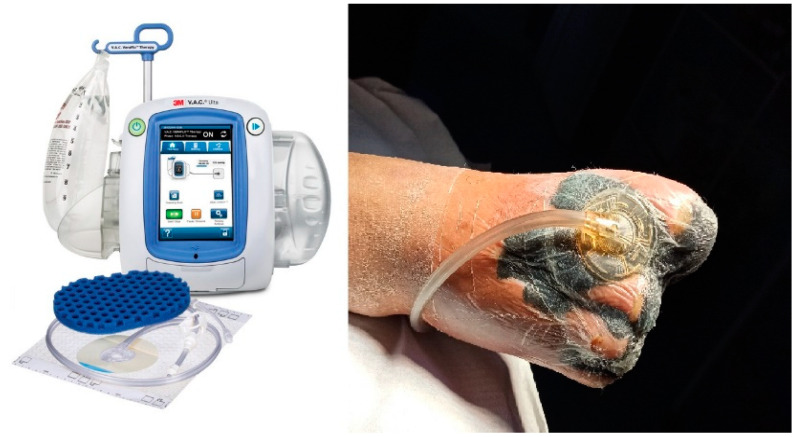
Applying the negative pressure wound therapy (NPWT) system using a sterile foam and transparent film dressing connected to a suction device set at –125 mmHg promotes exudate removal and tissue granulation in the wound bed.

To ensure the consistent and reliable tracking of symptom evolution and patient perception, the questionnaire was administered at three key time points: on Day 0, at hospital admission, before any specialized intervention; on Day 5, when the vacuum-assisted drainage system was removed and Spincare™ nanofiber application began (for the study group); and on Day 10, at discharge, to evaluate perceived improvement, comfort, and satisfaction with the treatment. This subjective evaluation tool provided valuable insights into the real-time impact of therapy beyond objective wound assessment and helped highlight the clinical benefits of Spincare from the patient’s perspective. After the wound bed was prepared on the first day of hospitalization, all patients received negative pressure wound therapy (NPWT). This consisted of applying a sterile foam and film dressing connected to a suction device that maintained a pressure of 125 mmHg, aiding in wound drainage and tissue granulation (Figure 1).

All patients followed the same supportive pharmacological regimen throughout their 10-day hospital stay, which included anti-inflammatory drugs, pain relievers, and vitamin supplements. No antibiotic therapy was required, as the wounds were not infected and microbiological cultures were negative.

### 2.3. Application of Spincare™ Nanofiber Therapy

Starting on Day 5, the treatment protocols diverged between the two groups after removing the vacuum-assisted drainage system. The control group received conventional local wound care, including daily cleansing with antiseptic solutions. In the control group, after the initial 5 days of negative pressure wound therapy (NPWT), standard wound care consisted of daily wound cleansing with a 0.05% chlorhexidine antiseptic solution, application of sterile non-adherent dressings (such as Atrauman^®^ Paul Hartmann AG, Heidenheim, Germany or equivalent), daily wound assessment and dressing changes, along with symptomatic pharmacologic management using anti-inflammatory and analgesic medications. No advanced wound healing interventions were utilized during this phase, such as growth factors, grafts, or nanomaterials. In contrast, patients in the study group received Spincare™ nanofiber therapy, a modern wound care technology based on electrospinning.

**Figure 2:**
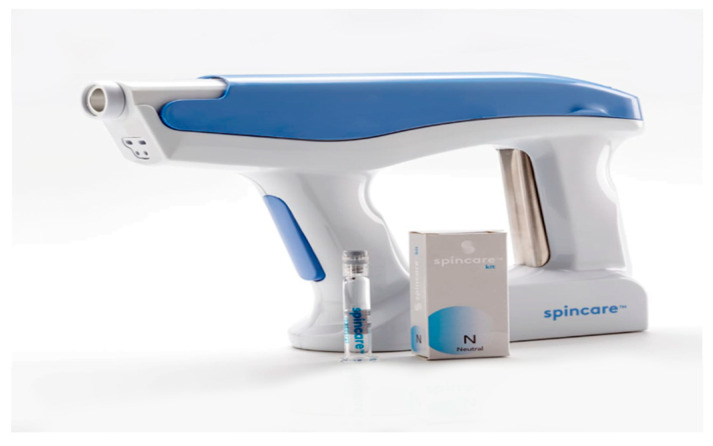
Handheld Spincare™ electrospinning device and polymer solution ampoule used to generate and deposit a nanofiber scaffold directly onto the wound surface in a clinical setting.

The procedure involved applying a sterile nanofiber matrix onto the wound surface using a portable electrospinning device (Figure 2). This handheld device generates a high-voltage electric field that transforms a liquid polymer solution into ultra-thin fibers deposited in situ to form a skin-like scaffold over the wound. The Spincare™ system utilizes a sterile, biocompatible medical-grade polyurethane-based polymer solution (specific formulation proprietary), electrospun into nanofibers with an average diameter of 400–800 nanometers. The resulting matrix exhibits a porosity greater than 80%, facilitating moisture balance and gas exchange. Electrospinning parameters include an applied voltage of 12–15 kV, a collector distance of approximately 10–12 cm, and ambient humidity between 40% and 60%. The resulting matrix mimics the structure of the extracellular matrix, promoting cellular proliferation, adhesion, and accelerated reepithelialization.

NPWT was applied for the first five days in both groups to optimize wound bed conditions. Spincare™ nanofiber therapy was initiated on Day 5 to enhance epithelialization, following guidelines that recommend advanced therapies be applied only after adequate wound bed preparation [5].

In both groups, skin biopsy samples were collected at two time points—upon admission and discharge—to evaluate the associated histopathological changes of each treatment. A 2 mm punch biopsy was performed in all cases. Samples were fixed in 10% buffered formalin (pH 7.4) for up to 48 h and processed automatically (Excelsior™ Epredia system) into paraffin-embedded blocks. Sections were stained using hematoxylin and eosin (H&E) and examined under a Leica DM 3000 LED microscope.

On average, the duration of Spincare™ therapy ranged from 10 to 21 days, depending on wound severity. Most patients required 1 to 3 applications of the nanofiber matrix, spaced approximately 7–10 days apart.

The Spincare™ nanofiber layer remained in place throughout the healing process, functioning as a temporary skin substitute. Depending on the wound progression, some patients required a second or third application before discharge or during follow-up visits. The dressing naturally detached as reepithelialization progressed, minimizing discomfort and eliminating the need for daily dressing changes.

## 3. Results

### 3.1. Demographics Characteristics

A total of 60 patients were enrolled in the study, with an approximately equal gender distribution between the two groups. In the control group (vacuum therapy), 53.3% of participants were female and 46.7% were male, while in the Spincare group, 56.6% were female and 43.3% were male, as shown in Table 1. Overall, a slight predominance of female patients was observed. The age distribution revealed that most participants (66.25%) were between 46 and 64 years of age, followed by those aged 65 years and older (11.25%), and a smaller proportion (22.5%) in the 26–45-year age range. All included patients met the study’s predefined eligibility criteria. Regarding the environment of origin, most patients (61.6%) resided in urban areas, compared to 38.7% from rural regions. This may reflect a higher rate of sedentary lifestyles or delayed access to early wound care interventions among urban populations, potentially contributing to the development of chronic, non-healing wounds. Regarding underlying pathologies, the three most common conditions associated with delayed wound healing were chronic obstructive arterial disease (COAD)—38.3% of patients; diabetes mellitus with complications—33.3%; and peripheral vascular disease (PVD)—28.3%.

The distribution of these conditions was relatively balanced between the two groups, ensuring the comparability of outcomes. Additionally, analysis of wound chronicity revealed that 65% of patients reported wound onset between 30 and 90 days before admission, while 35% presented with wounds persisting for more than 90 days.

**Figure 3:**
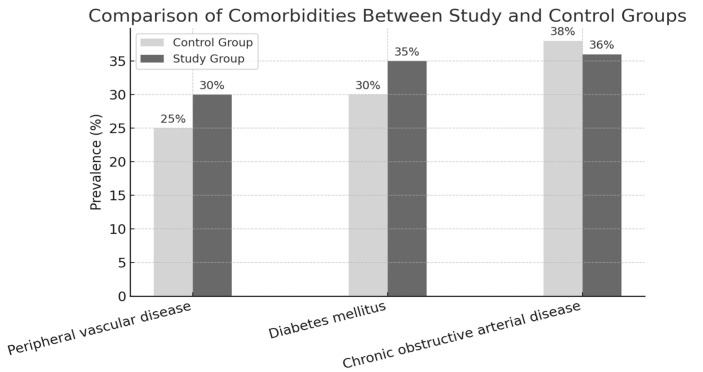
Background diseases.

To assess baseline comparability, we analyzed the distribution of key comorbidities associated with chronic wound development: chronic obstructive arterial disease (COAD), diabetes mellitus, and peripheral vascular disease (PVD). COAD was most common (38.3%), followed by diabetes (33.3%) and PVD (28.3%). Across groups, diabetes was equally represented (33.3%). COAD was slightly more prevalent in the control group (40% vs. 36.6%), while PVD was marginally higher in the study group (30% vs. 26.6%). The distribution of comorbidities between the two groups was comparable, indicating a balanced baseline and enabling a meaningful comparison of treatment outcomes, as shown in Figure 3.

Of the 60 patients enrolled, 65% reported that their wounds had been present for 30 to 90 days before admission. In contrast, 35% had wounds that had persisted for over 90 days, indicating a chronic and prolonged evolution before hospitalization.

### 3.2. Pain Scores

**Figure 4:**
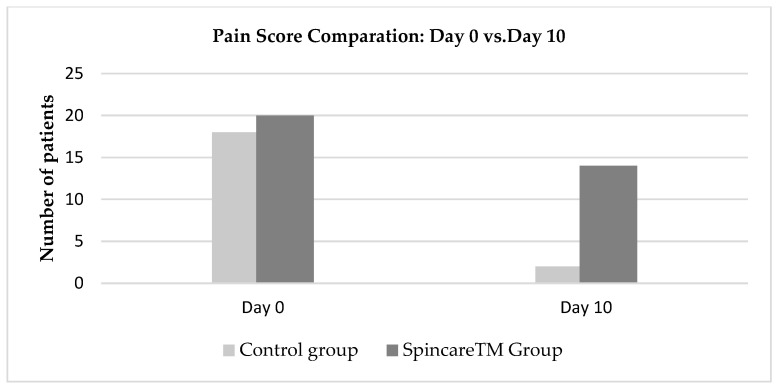
Evolution of pain scores from Day 0 to Day 10 for both the control and Spincare™ groups.

Pain was the most frequently reported symptom in 99% of all admitted patients. On Day 0, 20 out of 30 patients in the Spincare™ group and 18 out of 30 in the control group rated their pain as seven or higher on the numerical pain scale, where 10 indicates severe pain that limits mobility. By Day 10, a notable difference was observed between the two groups: in the Spincare™ group, 14 patients (46.6%) reported complete pain relief, assigning a score of 0 on the scale. In contrast, only two patients (6.6%) in the control group reported similar improvement (Figure 4).

Figure 4 presents the evolution of pain scores between Day 0 and Day 10 in both groups. Evolution of pain scores from Day 0 (admission) to Day 10 (discharge) in both treatment groups. Pain reduction is observed in the Spincare™ group compared to the control group. For detailed wound severity at baseline, refer to Table S1. At admission, the number of patients reporting severe pain (scores between 7 and 10) was similar: 20 in the Spincare™ group and 18 in the control group, confirming comparable baseline symptom severity.

By Day 10, the difference became evident. In the Spincare™ group, 14 patients reported complete pain relief (score 0), compared to only two patients in the control group. This suggests an apparent analgesic effect associated with the application of nanofibers.

The electrospun nanopolymer likely contributed to pain reduction by forming a protective layer that minimized local irritation, reduced the need for frequent dressing changes, and maintained a moist, healing-friendly environment.

Beyond clinical healing, this reduction in pain had a direct positive impact on patient comfort and emotional well-being, reinforcing the role of Spincare™ not only as an effective wound therapy but also as a patient-centered innovation in chronic wound care.

**Figure 5:**
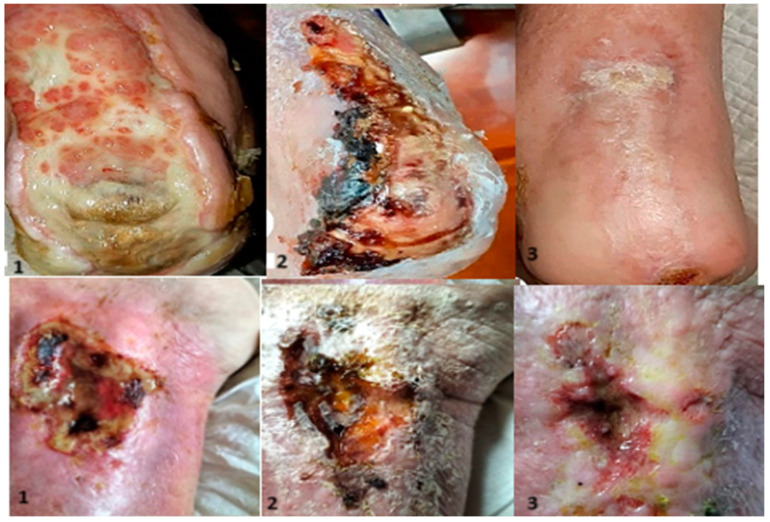
Evolution: 1—appearance at admission; 2—aspect after the first application; 3—appearance at the control.

**Figure 6:**
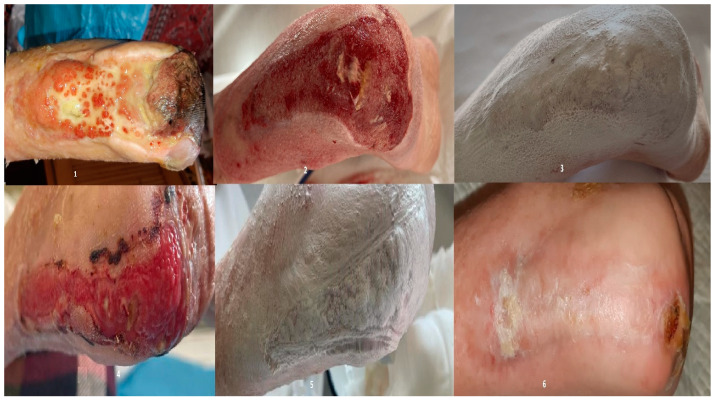
1—wound appearance at admission; 2—after vacuum therapy; granulation stage; 3—first nanofiber application; 4—appearance after first application; 5—appearance after first application; 6—wound aspect 21 days after the second application.

This newly studied therapy shows promising effects on wound healing and its influence on the patients’ overall condition (Figure 5 and Figure 6).

Patients from the Spincare™ group reported high satisfaction with the new therapy, primarily due to the improved comfort and freedom it provided. Many resumed their daily activities, including showers, thanks to the nano-polymer film’s water resistance and reduced need for frequent dressing changes. Compared to the control group, a significantly higher proportion of patients (46% vs. 23%) achieved the maximum satisfaction score at discharge, indicating a better perceived overall condition. Patient adaptation to therapy was evaluated between Days 5 and 10 after both groups had received the initial 5 days of standard vacuum therapy.

On Day 10: 26 patients in the Spincare™ group and 19 in the control group received 1 point on the subjective scale, indicating rapid reintegration into daily life and active mobility; 2 patients in the Spincare™ group and 8 in the control group received 2 points, reflecting delayed recovery and pain during walking beyond 50 m. A score of 3 points, indicating difficulty resuming daily activities, was given by two patients in the Spincare™ group and three in the control group.

These findings suggest that Spincare™ therapy had a positive impact on patient recovery and autonomy, improving not just clinical healing but also functional reintegration. Additionally, 27 patients in the Spincare™ group required a second nanofiber application at discharge, and 17 needed a third application, performed 10 days later or after the second layer began to degrade naturally. This layered approach allowed the dressing to remain in place during healing and minimized disruption to the wound site.

At 10 days post-discharge, patient follow-up revealed notable differences between the two groups regarding wound progression and treatment needs. In the control group, 18 patients required readmission to reevaluate the therapeutic approach. Nine patients continued antiseptic treatment, remaining in the granulation phase, while three showed clinical deterioration, presenting with multiple areas of necrosis.

**Figure 7:**
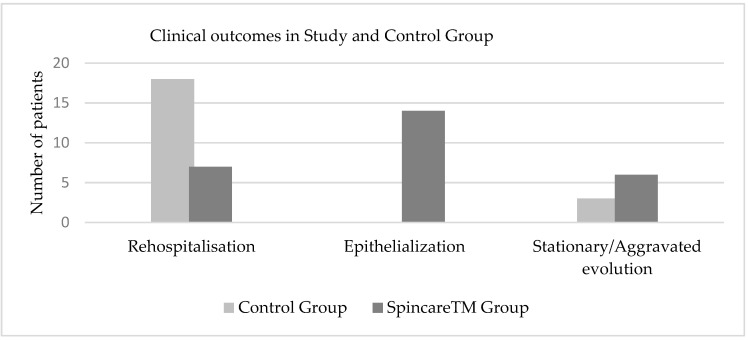
Comparative clinical outcomes of wound healing at 10 days post-discharge in both study groups, illustrating higher rates of epithelialization and reduced rehospitalization in the Spincare™ group.

In contrast, the Spincare™ group showed more favorable outcomes: 14 patients progressed to the epithelialization stage and did not require hospitalization; 2 patients had fully closed wounds; 7 patients were readmitted for scheduled nanofiber reapplication; 6 patients experienced wound deterioration with signs of superinfection, most likely due to poor hygiene or non-compliance with home care instructions; 1 patient did not return for follow up. Figure 7 illustrates the wound evolution and clinical outcomes at this post-discharge checkpoint.

The results presented in Table 2 emphasize the clear clinical advantage of Spincare™ nanofiber therapy over conventional treatment.

Nearly half of the patients in the Spincare group (46.6%) reached the epithelialization stage without requiring hospitalization, whereas none of the patients in the control group achieved this milestone. Additionally, two patients (6.6%) in the study group achieved complete wound closure, highlighting the regenerative potential of electrospun nanofibers, particularly in complex, chronic wounds. The need for readmission was substantially lower in the study group, with only 23.3% requiring nanofiber reapplication, compared to 60% in the control group, which required further therapeutic reassessment. This difference reflects not only faster healing but also a reduction in the healthcare burden associated with prolonged or ineffective wound care. The rate of complications, such as wound superinfection, was also lower in the Spincare™ group (20% vs. 30%), suggesting better control over local wound environments when using nanofiber dressings. Furthermore, fewer patients were lost to follow up in the Spincare™ group (1 vs. 3), which may indicate higher patient satisfaction and adherence.

Chi-square analyses revealed multiple statistically significant differences between the groups, including epithelialization (*p* = 0.00016), rehospitalization (*p* = 0.00007), and wound closure (*p* = 0.00068), reinforcing the effectiveness of Spincare™ in promoting healing and reducing complications.

These findings highlight the clinical benefits of electrospun nanofiber technology in chronic wound management, particularly its ability to support epithelial regeneration when post-discharge hygiene protocols are correctly followed.

## 4. Discussion

Chronic wounds represent a significant clinical and socio-economic burden worldwide, particularly in aging populations and individuals with comorbidities such as diabetes, peripheral arterial disease, and chronic venous insufficiency [2,5,7]. The failure of wounds to progress through the normal healing stages leads to prolonged morbidity, an increased risk of infection, and higher healthcare costs [3,7,14]. In this context, modern technologies such as electrospun nanofiber dressings are being increasingly explored for their ability to accelerate tissue regeneration and improve patient outcomes [10,11,12,13,14,15,16,17].

Our study used Spincare technology, based on in situ electrospinning, and significantly improved outcomes compared to conventional vacuum-assisted wound therapy. Notably, 46.6% of patients in the Spincare™ group reached the epithelialization stage without requiring readmission, while none in the control group achieved this level of healing. Additionally, 6.6% of patients in the study group had fully closed wounds, a remarkable outcome for chronic wounds [9,18,19,20,21].

Numerous studies have confirmed that electrospun nanofibers promote wound healing through their high porosity, mechanical adaptability, and bioactivity, supporting cellular migration, proliferation, and epithelialization. However, reproducibility remains a known limitation of electrospinning technologies due to their sensitivity to operational and environmental parameters [6,9,22].

Pain reduction was another key advantage. Patients in the Spincare™ group reported a significantly higher rate of complete pain relief by Day 10 (46.6%) compared to the control group (6.6%). This may be attributed to the protective, non-adherent properties of the nanopolymer film, which minimize dressing-related trauma and friction, consistent with findings from other clinical trials using nanofiber matrices [13,16,22,23,24].

From a statistical perspective, the Chi-square analysis revealed highly significant differences between the two groups across multiple clinical outcomes. For example, differences in epithelialization stage, rehospitalization rates, and wound closure rates showed *p*-values of less than 0.01, with the overall wound healing outcome reaching a *p*-value of 0.00016. This confirms the reliability of the observed improvements and supports the use of Spincare™ as a superior alternative in wound management [20,25,26,27,28,29].

The biological mechanism of action of electrospun nanofibers is based on their structural similarity to the extracellular matrix, which provides a scaffold that facilitates cell adhesion, proliferation, and migration [8,11,23]. These characteristics make nanofiber-based dressings particularly suitable for wounds with poor vascularization or delayed healing, such as those found in diabetic foot ulcers or those associated with arterial disease [4,14,28].

Patient adaptation and satisfaction were also notably improved in the study group. Thanks to the nanopolymer layer’s water-resistant properties, most patients could resume hygiene routines, including showering, without compromising wound protection [6,26]. Furthermore, rehospitalization rates were significantly lower in the Spincare group (23.3%) compared to the control group (60%), thereby reducing healthcare load and improving quality of life [25,27,30].

Despite these promising results, some patients in the Spincare™ group (20%) experienced wound worsening due to superinfection. These cases were strongly associated with non-compliance to home hygiene protocols and therapeutic instructions, highlighting the critical role of patient education and follow-up in maximizing treatment success [19,31].

Our findings are consistent with a growing body of evidence supporting nanotechnology-driven approaches to treating chronic wounds. Studies have shown that electrospun fibers can be functionalized with growth factors, antimicrobials, or drugs to enhance their bioactivity further [17,28,32].

Integrating Spincare™ into clinical practice significantly improves healing outcomes, pain control, and patient comfort, with statistically and clinically relevant benefits compared to standard care. This reinforces the role of electrospun nanofibers as a promising tool in the personalized management of chronic wounds.

The findings of this study demonstrate that Spincare™ electrospun nanofiber technology offers clear advantages in managing chronic wounds.

Although the study demonstrated early clinical benefits within the first 10 days, the short follow-up period is limited. A longer-term evaluation, over 30 to 60 days, is necessary to assess the durability of healing and potential recurrence, and such a follow-up study is currently planned.

Compared to conventional vacuum-assisted therapy, Spincare™ resulted in faster epithelialization, more significant pain reduction, lower rehospitalization rates, and improved patient satisfaction, all supported by statistically significant differences in clinical outcomes.

From an economic standpoint, although the Spincare™ electrospinning device’s initial cost is higher than that of standard NPWT materials, this is offset by a significantly lower need for hospital readmissions, reduced use of secondary dressings, and fewer daily interventions. Consequently, the total treatment cost per patient was comparable between the two groups. This makes Spincare™ a potentially accessible solution for healthcare systems prioritizing efficiency and outpatient care.

The structural and functional properties of electrospun nanofibers, which closely resemble the extracellular matrix, provide an optimal healing environment by promoting cellular regeneration while reducing mechanical irritation and the need for frequent dressing changes. These benefits were especially notable in patients with comorbidities, who often present with impaired wound healing.

Importantly, the success of this technology is closely tied to patients’ proper adherence to post-discharge hygiene protocols. Where compliance was ensured, Spincare™ facilitated superior wound progression and minimized complications.

Several other limitations must be acknowledged beyond the short follow-up period already discussed. The relatively small sample size may affect the generalizability of results to broader populations and various wound types. The lack of blinding and objective assessment tools for pain and comfort also introduces a potential risk of bias in patient-reported outcomes. The study also lacked molecular-level evaluations, such as analysis of cytokines or angiogenic factors, which could have provided deeper mechanistic insights into the effects of nanofiber therapy. Future studies should address these limitations by incorporating larger, multicenter cohorts, extended monitoring, and comprehensive biomolecular analyses.

## 5. Conclusions

Spincare™ electrospun nanofiber therapy significantly improves healing outcomes, reduces pain, and enhances patient comfort compared to conventional wound care.

Its biomimetic structure supports tissue regeneration, while the non-invasive application offers practical advantages in clinical use. When accompanied by proper hygiene compliance, Spincare™ is an effective and innovative tool in the personalized management of chronic wounds.

## Figures and Tables

**Table 1:** Patient demographic characteristics at the baseline visit.

Characteristics at Baseline Visit	N (%)
Gender	
Females	55 (71.25)
Males	45 (28.75)
Age	
65	42 (11.25)
46–64	16 (66.25)
26–45	2 (22.5)
Environment of origin	
Rural	24 (38.70)
Urban	36 (61.60)

**Table 2:** Wound healing outcomes at 10 Days post-discharge with statistical significance.

Outcome	Study Group (n = 30)	Control Group (n = 30)	Study Group (%)	Control Group (%)	*p*-Value
Epithelialization stage (no hospitalization)	14	0	46.66	0.00	0.00007
Wound closed	2	0	6.66	0.00	0.47202
Rehospitalization for nanofiber reapplication	7	18	23.33	60.00	0.00883
Worsened stage (superinfection)	6	9	20.00	30.00	0.55098
Did not show up for the follow up	1	3	3.33	10.00	0.60477

*p*-values < 0.05 are considered statistically significant.

## Data Availability

The original contributions presented in this study are included in the article. For further inquiries, please contact the corresponding authors.

## Supplementary Materials

The following supporting information can be downloaded at: https://www.mdpi.com/article/10.3390/bioengineering12050500/s1, Table S1. Baseline wound area and depth for each patient in the study and control groups.
