# Supplementary material for: Electrospun Nanofibers in Wound Healing: Real-World Evaluation of Spincare™ Technology
Source: Bioengineering (Basel). 2025 May 9;12(5):500. doi: 10.3390/bioengineering12050500 (PMC12109364; doi:10.3390/bioengineering12050500)
Supplement: Supplementary file 1 [file bioengineering-12-00500-s001.zip › bioengineering-3592719-supplementary.pdf]

Table S1. Baseline Wound Area and Depth for Each Patient in the Study and Control Groups

Patient ID	Treatment Group	Wound Area (cm^2)	Wound Depth (cm)
PAC-001	Spincare	20.9	0.37
PAC-002	Spincare	21.4	0.52
PAC-003	Spincare	20.2	0.98
PAC-004	Spincare	17.1	0.67
PAC-005	Spincare	6.7	0.37
PAC-006	Spincare	22.2	0.58
PAC-007	Spincare	25	0.77
PAC-008	Spincare	20.8	0.63
PAC-009	Spincare	24.9	1.31
PAC-010	Spincare	15.2	0.42
PAC-011	Spincare	22.4	0.83
PAC-012	Spincare	11.6	0.49
PAC-013	Spincare	14.1	0.9
PAC-014	Spincare	9.9	0.9
PAC-015	Spincare	17	0.61
PAC-016	Spincare	22.9	1.41
PAC-017	Spincare	24.2	1.05
PAC-018	Spincare	11.7	0.56
PAC-019	Spincare	11.5	0.72
PAC-020	Spincare	23.7	0.41
PAC-021	Spincare	13.5	1.36
PAC-022	Spincare	7.6	0.73
PAC-023	Spincare	11	0.78
PAC-024	Spincare	10.6	1.39
PAC-025	Spincare	12	1.04

PAC-026	Spincare	24.8	0.9
PAC-027	Spincare	24	0.24
PAC-028	Spincare	20.1	0.61
PAC-029	Spincare	6.3	0.82
PAC-030	Spincare	20.4	1.3
PAC-031	Control	11.9	0.21
